# Randomised clinical trials in surgery: are we at a crossroads?

**DOI:** 10.1097/MS9.0000000000001457

**Published:** 2023-11-07

**Authors:** Tetraj P. Ramchurn, Samiran Nundy

**Affiliations:** Department of Surgical Gastroenterology and Liver Transplant, Sir Ganga Ram Hospital, New Delhi, India

The randomized clinical trial is a prospective study design that measures the effectiveness of a new intervention or treatment. It is the most meticulous and robust research method for establishing whether a cause–effect relationship exists between an intervention and a measured outcome. Three different types of surgical trials have been described: Type 1 trials compare medical management in surgery; Type 2 trials compare surgical techniques; and Type 3 trials compare surgical and nonsurgical interventions^1^. A surgical intervention is defined as one that involves physically changing body tissues and organs through manual operations such as cutting, suturing, abrading, or the use of lasers^2^. Surgical interventions are complex and involve multiple factors that act either independently or inter-dependently to influence outcomes^3^. Thus, a surgical trial is fundamentally different from a pharmacological trial in as much as a surgical intervention is different from a ʻpillʼ. Although they represent the best level of evidence, randomised trials in surgery are uncommon^4^ and only about 15% of published RCTs are related to surgical interventions^5^.

## Tenets of randomised clinical trials

The design and conduct of a randomised clinical trial are based on certain basic principles. Generally, the need for an RCT in surgery arises when peer experts in a particular field identify gaps in knowledge gathered through observational (case–control and cohort) studies and reach equipoise over the efficacy or effectiveness of an intervention or treatment. Then a research question is usually framed in the ʻPICOʼ format^6^ defining the patient and comparator group, the intervention, the measurable outcomes and the clinical end-points. A research protocol is subsequently designed whereby various aspects like the target population, process of randomisation, blinding of intervention, treatment guidelines, outcome definitions, sample size required, ethical approval, informed consent process, and data analysis methodology are clearly defined. In order to ensure the high-quality of the RCT, an expert statistician is often consulted regarding the calculation of the sample size and the power of the study. Once the protocol is approved by an independent ethics committee, it is now compulsory that RCT protocols are either registered with a publicly available trial registry or published in an indexed medical journal. Additionally, the International Committee of Medical Journal Editors (ICMJE) has recently recommended that clinical trials that will begin enroling participants on or after 1st January 2019 must include a data sharing plan when registering the trial^7^. An independent data monitoring committee is usually established to periodically oversee the progress of the clinical trial by doing an interim analysis. When the study reaches its predetermined accrual, the data are ideally analysed based on an intention to treat approach and the outcomes are reported as per the CONSORT guidelines^8^.

The Randomised Clinical Trial occupies the pre-eminent position among the hierarchy of evidence^9^. A well-designed RCT yields reliable results, which represent the highest level of evidence. As the paradigm has shifted to evidence-based practice, RCT study designs have become more popular. It is recommended that clinical practice decisions are based on evidence gathered from well-conducted RCTs^10^. Various clinical guidelines like the ESMO (European Society for Medical Oncology) and the USA’s NCCN (National Comprehensive Cancer Network) guidelines for management of cancer and quality assurance bodies like the USFDA (United States Food and Drug Administration) support their recommendations and drug and medical device approval decisions, respectively, based on scientific evidence gathered through RCTs. The reliance on RCTs for clinical decision-making is explained by certain unique methodology aspects inbuilt in the RCT study design. Data collection is *prospective* in nature and therefore offers scope for more accurate and complete data collection and diminishes the potential for sources of bias namely in the recall and selection process. *Randomisation* is another cornerstone of RCT^11^. Through randomisation, selection and participation bias are mitigated and the effects of confounding factors on outcome are addressed. This is because the act of randomisation distributes participants’ variables (both observed and unobserved) between the study groups, such that the groups become comparable and any differences in outcome will then be due to the intervention only. This is not possible with any other study design. *Blinding* is another hallmark of RCT design, which effectively reduces participant as well as observer bias. Last but not least, the rigour in the design and conduct of the RCT, from protocol writing through ethical committee approval to statistical analysis and reporting, ensures transparency, standardisation, safety of intervention, and generalisability of outcome and thereby guarantees the excellent quality of the evidence derived.

## Evolution of randomised clinical trials in surgery

For centuries, surgeons have, through perseverance and passion for research, played key roles in the development of surgical science and evidence-based medicine. As early as the 16th century, the French surgeon Ambroise Pare had conducted a study on wound healing where he compared the effect of elderberry oil versus an egg yolk concoction and he documented the outcome. Then, in the 18th century, the Scottish surgeon, James Lind designed a prospective controlled study of six possible remedies for preventing scurvy in sailors and he concluded that citrus fruits performed best^12^. The trials and tribulations of surgeons continued through the 19th century where they started vouching for statistical analysis and began quantifying outcomes of amputations, surgical drainage of empyema, and tracheostomy for croup^13^. In the late 19th century, the development of anaesthesia and the implementation of antisepsis and asepsis ushered in the concept of hospitalisation of patients for surgery. As more patients underwent and survived hospital surgery, surgeons increased the volume and introduced innovative procedures. They started to collect data and report case series sometimes with a view to promote and advertise their skills and experience in a particular procedure. At the dawn of the 20th century, surgeons began to understand concepts like bias, generalisability of trial results and therefore improved from the alternate allocation study method to randomisation and increasingly started to standardise the surgical intervention. Thereafter, in the 1940s–1950s, several landmark surgical RCTs with large sample size began to be reported^13^. The Helsinki Declaration of 1964 urged for further refinements in the RCT study design in the areas of ethics in medical research and informed consent prior to an intervention. By the 1980s, surgeons had widely adopted RCTs as the gold standard for surgical research^13^. However, with the advent of industrialisation and automation, there has been a gradual historical shift in global disease burden from acute and infective pathologies to lifestyle related chronic diseases. In this changing landscape, is the RCT research model still relevant?

## RCT’s in surgery. Why are there so few?

A 2003 review of the five leading surgical journals found that only 3.4% of published articles were RCTs^5^ and a 2021 systematic review of surgical trials published in the two highest impact factor journals in six key surgical specialties between 2008 and 2020 showed that the average number of published trials per year did not increase during the study period (average 32 per year)^14^. To improve the quality of RCTs in surgery, the CONSORT (Consolidated Standards of Reporting Trials) guidelines were developed in 1996^15^ and revised in 2010^16^. However, compliance to CONSORT guidelines remains a perennial issue and recent data show that improvements in the design, implementation, and reporting of randomised clinical trial in surgery are warranted^14^. As opposed to pharmaceutical RCTs, the methodological design and conduct of the surgical RCT pose practical and fundamental difficulties to surgeons and participants.

- The surgical intervention is an irreversible act and leaving the surgical decision to chance through randomisation, makes patient recruitment a challenge.
- Blinding of the investigator-surgeon is practically impossible and if subject assignment is known to the surgeon, bias can be introduced because extra care may be given to the intervention procedure which the surgeon is willing to promote^15^. Viewed through the lens of medical ethics, the acts of randomisation, concealment and blinding defy the basic tenets of autonomy and nonmaleficence. Thus, finding the fine balance between the principles of clinical research and the principles of clinical practice is often the challenge.
- There are several surgeon centred issues that explain the paucity of surgical trials. Most surgical procedures now in practice have not been supported by an RCT. There are no pressures from governments or third-party payers for surgical innovations to be validated by RCTs. Surgical trials are deployed by surgeons out of their own sense of scientific and professional obligation.
- Conducting RCT is labour intensive, time-consuming, and expensive. In addition to these concerns, the surgeon-investigator is faced with fierce criticisms from peers for challenging the lore while simultaneously grappling with the rigour of the RCT study requirements.
- Standardisation of the intervention and generalisability of the results are two other pressing issues of surgical RCTs and as it is said ʻunlike 20 milligram tablets, no two surgical procedures are the sameʼ^17^. Standardisation in surgical trials means that the participating surgeons have to be at the same point on the learning curve. This requires the surgeons to unlearn, learn, and relearn new skills and this process is costly and time-consuming. However, despite all efforts to bring all the participating surgeons to the same level of proficiency the questions of generalisability will persist. Operations are complex interventions, with outcomes depending not just on the surgeon and procedure, but also on the patient’s anatomy and pathology; the surgical team of nurses, assistants, and anaesthetists; postoperative management; and rehabilitation programs^18^.

## Delivering randomised clinical trials in developing countries

Around 83% of the world population live in developing countries and these countries bear the greatest burden of diseases. Yet, less than 10% of health research funds are devoted to solving health problems indigenous to developing countries^19^ and less than 20% of clinical trials are conducted in these countries^20^. Paradoxically, these resource-limited nations are most in need of high-quality research to prioritise the allocation of their scarce resources^21^. While these nations have enormous research potential in terms of reduced cost and time to recruit participants^22,23^, they are conversely confronted with many barriers to the delivery of impactful randomised clinical trials. Some of the challenges facing researchers in developing countries are: lack of funding^24–35^, lack of skilled personnel^24,26,28,29^, complex and strict ethical and regulatory frameworks^32,35,36^, lack of research environment^29,34^, and competing demands (lack of time) of health professionals^24,29,32^. In such a scenario, conducting good-quality RCTs is very challenging and can result in wastage of already scarce resources. Hence, other research study designs must be explored and national as well as international collaboration must be sought in order to foster high-quality research by developing countries and for developing countries.

## Ground-breaking RCTs in Surgery

Undeniably, RCTs in surgery have contributed to the development of safer and more cost-effective surgical practices and have improved the quality of life and survival of many patients worldwide. This holds true in all the specialities of surgery: General surgery, Orthopaedics, Plastic surgery, Urology, Neurosurgery, Cardiac surgery, Surgical oncology and Gynaecology. The 20-year follow-up of a randomised trial comparing total mastectomy, lumpectomy and lumpectomy plus irradiation for the treatment of invasive breast cancer by Fisher *et al*.^37^ was instrumental in the paradigm shift to breast conserving surgery for breast cancer. Another landmark RCT in surgery is the STICH trial^38^, a negative RCT conducted in the early 2000s, which demonstrated that surgical revascularisation in patients with ischaemic heart failure did not improve survival or reduce hospitalisation rates when compared to medical therapy alone. In the field of urology, influential RCT studies on minimally invasive prostatectomy vs open radical prostatectomy and RCT studies on open donor nephrectomy vs laparoscopic donor nephrectomy, phased out old practices and shaped clinical practice forever. So far, randomised clinical trials in surgery have been delivering but now with growing healthcare problems in epidemic proportions, the traditional RCT design may require to evolve.

## The way forward

Very often, we observe that well-designed RCTs in surgery fail to convince the surgical society for the reason that the sample size is not adequate or the study is conducted by a single surgeon or a single institution and hence the results are not generalisable. Additionally, the rapid pace of technological innovations and occult market forces are increasingly leading to a situation where novel technologies and techniques in surgery are becoming mainstream long before they are validated by good-quality scientific data. The randomised controlled trial as a research tool may not be agile and resilient enough to adapt to the rapid dissemination of disruptive technology. However, technological advances can be leveraged to benefit the surgeon-researcher. With the advent of blockchain technology and broadband internet with 4G, 5G, and 6G technology, large amount of medical health records can now be safely transferred and accessed from any corner of the globe. This infrastructure can facilitate collaborative networks whereby like-minded scientists can contribute and successfully deliver high-quality randomised controlled trials in surgery. The UK boasts of such a collaborative network. It was initiated by the Royal College of Surgeons of England^39^ and within only 3 years there was an increase in the number of hospitals recruiting into surgical studies and the number of patients entering surgical RCTs doubled (11 000 in 2011–2012 to 25 500 in 2014–2015)^40^. Similar models of Study Groups exist in countries like the Netherlands and Japan and generally their RCTs are regarded in high esteem. In the same vein, technologically driven global collaborative efforts among surgeons may be the solution to all the obstacles that have hitherto come in the way of RCTs in surgery.

Furthermore, the surgical society at large must accept the fact that RCTs have limitations and that there are other alternate study designs that can provide valid evidence for clinical and public health decision-making. In the hierarchy of the level of evidence, a fine straight line demarcates one study design from the other type. This notion has been challenged in a scientific paper by Murad *et al*.^10^ in which the author introduced the idea that the demarcating lines across the pyramid of evidence may in fact be ‘wavy’. It recognised the fact that data from a non-RCT may sometimes be of better quality than that derived from a poorly designed RCT. Large nonrandomised observational studies, nation-wide registry database for rare diseases and propensity-matched comparative studies are potentially highly valuable sources of data and must not be brushed under the carpet at the expense of RCT derived data. In this era of Big Data, Supercomputing, Machine Learning and Artificial Intelligence, large datasets can be mined and analysed for finding real-time solutions. For instance, by matching the demographic profile and clinical variables of a patient and the disease biologic profile with a similar patient across the globe, a precise and personalised treatment plan with prognostication can be provided. The ORIEN (Oncology Research Information Exchange Network) is one such platform in the United States whereby 19 member Cancer Centres longitudinally collect and share clinical data with a view to provide individualised cancer care solutions. In fact, the need of the hour is for transparent and painstaking medical record keeping and data sharing on secured global collaborative digital platforms. The idea is to shift from decision-making based on the past experience of a particular group towards action based on the best current available medical evidence. The goal must be actionable data – data that are objective and can substantiate for clinical and public health decisions and recommendations^41^.

Finally, developing secure and accessible data sharing platforms at a global level remains a challenge. These will require a comprehensive approach that encompasses both technical and organisational aspects, which include establishing clear data governance and policies, data classification, encryption, and masking and anonymisation but most importantly end-user education and training. It is vital to strike a balance between enabling data access for legitimate purposes and safeguarding data against unauthorised access and breaches through regular reviewing and enhancing security measures. Researchers in low-resource settings can benefit from online data collection tools like the REDCap (Research Electronic Data Capture), which is a secure web application for building and managing online surveys and databases. This would enhance the transparency and authenticity of their data. Global networks and funding agencies can collaborate with centres in the developing world to improve research protocols and standardise the medical management of surgical interventions. The tools are available. It is about time we innovate and embrace technological disruptions to our advantage.

## Ethical approval

Not applicable.

## Consent

Not applicable.

## Sources of funding

Not applicable.

## Author contribution

Dr T.P.R: corresponding author: conceptualisation and draughting; Prof S.N.: supervising and revising the draft.

## Conflicts of interest disclosure

Not applicable.

## Research registration unique identifying number (UIN)

Not applicable.

## Guarantor

Dr Tetraj Panray Ramchurn and Prof Samiran Nundy.

## Provenance and peer review

Paper was not invited.

## Data availability statement

No research data in this paper.
